# Sorghum Landrace Collections from Cooler Regions of the World Exhibit Magnificent Genetic Differentiation and Early Season Cold Tolerance

**DOI:** 10.3389/fpls.2017.00756

**Published:** 2017-05-09

**Authors:** Frank Maulana, Dilooshi Weerasooriya, Tesfaye Tesso

**Affiliations:** Department of Agronomy, Kansas State University, ManhattanKS, USA

**Keywords:** cold temperature, sorghum accessions, simple sequence repeat (SSR) markers, quantitative trait loci (QTL), marker-trait associations

## Abstract

Cold temperature is an important abiotic stress affecting sorghum production in temperate regions. It reduces seed germination, seedling emergence and seedling vigor thus limiting the production of the crop both temporally and spatially. The objectives of this study were (1) to assess early season cold temperature stress response of sorghum germplasm from cooler environments and identify sources of tolerance for use in breeding programs, (2) to determine population structure and marker-trait association among these germplasms for eventual development of marker tools for improving cold tolerance. A total of 136 sorghum accessions from cooler regions of the world were phenotyped for seedling growth characteristics under cold temperature imposed through early planting. The accessions were genotyped using 67 simple sequence repeats markers spanning all ten linkage groups of sorghum, of which 50 highly polymorphic markers were used in the analysis. Genetic diversity and population structure analyses sorted the population into four subpopulations. Several accessions distributed in all subpopulations showed either better or comparable level of tolerance to the standard cold tolerance source, Shan qui red. Association analysis between the markers and seedling traits identified markers *Xtxp*34, *Xtxp*88, and *Xtxp*319 as associated with seedling emergence, *Xtxp*211 and *Xtxp*304 with seedling dry weight, and *Xtxp*20 with seedling height. The markers were detected on chromosomes previously found to harbor QTLs associated with cold tolerance in sorghum. Once validated these may serve as genomic tools in marker-assisted breeding or for screening larger pool of genotypes to identify additional sources of cold tolerance.

## Introduction

Sorghum [*Sorghum bicolor* (L.) Moench] is the fifth most widely cultivated food and feed grain. While it is primarily used as animal feed in the developed world, sorghum is mainly used as a staple cereal in the Sahelian region of Africa and south Asia. Moreover, the use of the crop as industrial raw material is on the rise with substantial amount of sorghum grain going to brewery and biofuel industries.

Owing to its evolution under warm and dry conditions, sorghum has remarkable tolerance to drought and high temperature stresses compared to many other crops. Perhaps for the same reason, the crop is very sensitive to cold temperature and this is a major limitation to sorghum production in temperate regions (Alegre de la Soujeole and Miller, 1984). Cold temperature reduces seed germination and emergence, seedling growth and vigor (Yu and Tuinstra, 2001; Maulana and Tesso, 2013). The stress limits sorghum production both temporally and spatially by limiting the planting window in places where the crop is already under cultivation and through hampering its spatial expansion to colder regions. Options to address this situation are limited; one possible avenue is through developing and deploying hybrids with increased tolerance to cold temperature.

Sorghum germplasm from cold regions of the world such as the kaoliangs of China have shown to have higher seedling emergence and vigor under cold temperature compared to most traditionally grown cultivars (Singh, 1985; Cisse and Ejeta, 2003; Franks et al., 2006). However, the apparent agronomic inferiority and the high levels of tannin in this group of germplasm have limited their use in breeding programs. Therefore, there is an increasing need for alternative sources of cold tolerance in tannin free backgrounds for use in breeding programs. Although sorghum is a warm season tropical crop, it displays remarkable variability for every trait and magnificent ecological plasticity (Punguluri and Kumar, 2013) including adaptation to temperate production conditions. Thus it should be possible to find sources of cold tolerance elsewhere besides the Chinese kaoliangs where the cold tolerance trait appears to be highly correlated with high tannin concentration. In Africa, while the crop is predominantly grown under warm and dry conditions, variants adapted to cooler climates, such as the high altitudes of east Africa, may offer sources of tolerance to cold temperature. In Ethiopia, part of the vast north east Africa region where the crop is believed to have originated, sorghum is grown across a range of agro-ecologies stretching from the warm and dry lowlands to the cooler highlands situated at 2500 m above sea level. Depending on the regions, the temperature can range from as low as 8°C in the high altitudes to a high of about 38°C in the lowlands at least during part of the growing seasons. Moreover, sorghum is grown to a limited extent in other colder regions of the world such as Russia, Korea, Japan, Hungary, Turkey, etc. In addition to the ongoing efforts that seek to exploit the Chinese germplasm sources, assembling a population from other regions and screening them under cold temperature stress may lead to identification of new sources of cold tolerance that can be used in breeding programs. Such population can also be a useful resource for genetic mapping and development of molecular marker tools that may be used in marker-assisted breeding as well as for screening larger pool of genotypes to identify additional sources of cold tolerance.

A number of marker studies have been carried out in sorghum both to understand the molecular basis of certain traits such as plant height, flowering (Lin et al., 1995; Ulanch et al., 1996) and to develop maker tools for economically important traits such as drought tolerance and *Striga* resistance (Xu et al., 2000; Ejeta, 2007). Efforts to develop marker tools for cold tolerance in sorghum based on Chinese cold tolerance sources has achieved some success in that QTLs associated with improved cold temperature response have been identified (Knoll and Ejeta, 2008; Knoll et al., 2008; Burrow et al., 2011). However, their application in practical plant breeding was frustrated by the fact that some of the key QTLs detected for cold tolerance strictly overlapped with tannin QTLs (Wu et al., 2012). Though, it is not yet clear whether tannins actually condition improved cold tolerance, the fact that there is tight linkage has undermined the use of these markers in breeding programs to breed food/feed grain sorghums. Further marker study is needed to untangle this phenomena and chart ways for manipulating the trait. The completion of the sequencing of sorghum reference genome (Paterson et al., 2009) has opened new opportunities for studying complex problems like this and also for understanding structural, evolutional and functional genomics of sorghum. The fact that it has a small genome size of ∼730 Mb makes it a model species for genetic and genomic studies of cereals. However, those markers were developed on bi-parental mapping populations derived from crosses involving Chinese germplasm as source of cold tolerance. Due to the concern that cold tolerance QTL from these backgrounds may be linked to high tannin trait, the use of these markers in breeding programs may complicate development of tannin free feed/food grain sorghums.

Tannins, despite their health benefits have been reported to bind proteins and reduce its availability rendering high tannin grains unsuitable for animal feed or human food. However, there is broad opportunity to identify similar QTLs from tannin free sources. In the current study we assembled sorghum genotypes from colder regions of the world with the goal of screening and identifying tannin free sources of cold tolerance in breeding program. Although the number of markers used in genotyping the population was not large enough, we attempted to run association analysis to see if any of these markers are linked to genes mediating improved cold tolerance response.

Hence, the objectives of this study were (1) to assess early-season cold temperature stress response of sorghum germplasm from cooler environments and identify sources of tolerance for use in breeding programs, (2) to determine population structure and marker-trait associations among these germplasms for eventual development of marker tools for improving cold tolerance.

## Materials and Methods

### Genetic Materials and Phenotyping for Cold Tolerance

One hundred and thirty-six sorghum accessions originated in the temperate and sub-tropical regions of the world were included in this study. The list of accessions and countries of origin are presented in Supplementary Table 1. The accessions were selected and acquired through the USDA Germplasm Resource Information Network (GRIN). In order to maximize chances of identifying new sources of early-season cold tolerance, sampling of the accessions was purposely focused on materials from colder regions of the world. Seven photoperiod insensitive genotypes of tropical origin were also included for comparison. Cold tolerance check SQR from China and susceptible check SRN39 from Africa were included. The accessions were acquired in spring 2009 and seeds were increased during the 2009 summer season.

The study was conducted during the 2010 and 2011 seasons at the KSU agronomy research farm near Manhattan using a randomized complete block design in three replications. The plots were 5 m long single row spaced at 0.75 m between plots. The cold stress was imposed through early planting on 12 and 17 April in 2010 and 2011 seasons, respectively. The max/min soil temperature at 5-cm depth during early planting time was 20.0/13.1°C and 20.2/9.1°C (day/night) in 2010, 2011 seasons, respectively. Normal planting was done on 26 May and 9 June for the 2010 and 2011 seasons, respectively. The max/min soil temperature at 5-cm depth during normal planting was 29.3/17.2°C in 2010 and 32.1/19.4°C in 2011. Prior to planting, seeds were cleaned and surface-sterilized using standard sorghum seed treatment protocol (a mixture of Maxim 4FS, Apron XL, Concept III and colorant). At planting, 50 seeds from each accession were drilled into each plot using a cone planter adjusted to spread the seeds along the 5 m long plot.

Data were collected on seedling emergence, seedling vigor, seedling height and seedling dry weight. Seedling emergence data was recorded as the number of seeds with coleoptiles visibly emerged above ground 14 days after planting. Seedling vigor scores, seedling height and seedling dry weight were recorded on day 28 after planting. Seedling vigor was rated using a visual score on a 1 to 5 scale, with 1 = excellent and 5 = poor, as described by Maiti et al. (1981). This rating was based on the size and physical appearance of the seedlings. Seedling height was determined as the mean length of ten random seedlings in a plot measured from the base to the tip of the most top leaf. For seedling dry weight, ten randomly selected plants were harvested in each plot and the average weights recorded after the samples were dried at 120°C for 5 days.

Prior to analysis, all phenotypic data were checked for normality (Supplementary Figures 1–3) and traits that were not normal were transformed using arcsine square root transformation. Phenotypic data analysis was performed using SAS 9.1 (SAS Institute, 2003). The general linear model (GLM) procedure in SAS was used to estimate variation among genotypes, and all factors were treated as random effects. Mean comparisons for each parameter were performed using the Fisher’s Least Significance Difference (LSD) test at α = 0.05. Correlations of all traits within and between planting times were calculated. Broad-sense heritability estimates for all traits measured under both early and normal planting were calculated following the formula by Hallauer et al. (2010): *H*^2^ = *σ*^2^_g_*/[σ*^2^_g_ *+ σ*^2^_ge_*/e + σ*^2^_e_*/er]*; where *σ*^2^_g_ is the genetic variance, *σ*^2^_ge_ is the genotype-by-environment interaction variance, *σ*^2^_e_ is the residue variance, while *r* and *e* are number of replications and environments, respectively.

### Genomic DNA Extraction and PCR Assay

Fresh leaf tissues from young seedlings were harvested, freeze dried (ThermoSavant, Holbrook, NY, USA) and pulverized using a tissue grinder (Retsch, Newtown, PA, USA). The DNA extraction was performed using a modified CTAB procedure (Saghai-Maroof et al., 1984). The DNA pellets were washed with ethyl alcohol, air-dried and suspended in 200 μl Tris/EDTA (0.1TE) buffer. The quality and concentration of the DNA samples were determined using ND-1000 Spectrophotometer (NanoDrop Technologies, Inc, Wilmington, DE, USA).

Simple sequence repeat (SSR) markers of known map locations obtained from published literatures (Knoll and Ejeta, 2008; Knoll et al., 2008; Wang et al., 2009; Burrow et al., 2011; Gamar et al., 2013) were assembled. The markers were evenly distributed throughout the ten linkage groups of sorghum. The list of markers and their common characteristics is presented in Supplementary Table 2.

The PCR reactions were conducted using a PTC-225 Peltier Thermal Cycler (MJ Research, Inc, Watertown, MA, USA) with a total reaction volume of 30 μl. The reaction mixture contained approximately 30 ng genomic DNA, 5 *p*mole of each primer, 0.2 nM of each dNTP, 3.0 μl 10x- *Taq* buffer, 0.75 units *Taq* polymerase, 2.5 mM MgCl_2_, and 1 Mm Cresol red for tracking the fragments in the gel. The PCR amplification was performed using a touchdown procedure with reaction conditions as described by Taramino and Tingey (1996). The amplification products were differentiated by electrophoresis using 3% high resolution Metaphor agarose gels at 5V/cm in TBE buffer for 6 h. The DNA was stained with 0.015% ethidium bromide (EtBr) and the bands were visualized using a Gel Doc^TM^ documentation system under UV light. Quantity One Software (Bio-Rad, 1998) was used for scanning the gel and scoring the band sizes. Allele sizes produced from each accession were determined by comparing with a molecular weight hyperladder IV (Bioline USA Inc.).

### Genetic Diversity and Population Structure Analyses

The markers were first evaluated independently for polymorphism. Out of 67 markers screened, 50 of them produced sufficient level of polymorphism while the remaining 17 markers did not sufficiently distinguish the genotypes and hence were excluded from the analysis. The diversity analysis was performed using Nei’s 1983 method in PowerMarker version 3.25 (Liu and Muse, 2005) and confirmed in POPGENE 1.32 (Yeh et al., 1997). Diversity indices including total number of alleles, allele number per locus, gene diversity (expected heterozygosity), and polymorphism information content (PIC) were determined. The neighbor-joining (NJ) tree analysis was conducted with 100 replications of bootstrapping as implemented in PowerMarker (Liu and Muse, 2005) and the tree was constructed using Molecular Evolutionary Genetics Analysis (MEGA) 5.10 (Tamura et al., 2011).

The software STRUCTURE version 2.3.4 (Falush et al., 2012) was used to analyze the population structure based on admixture model where each individual draws some fraction of its genome from each of the *k* populations. It identifies gene flow events between subpopulations and individuals whose genotypes indicate admixture are assigned to two or more subpopulations. The STRUCTURE program was run 20 times for each subpopulation (*k*) value ranging from 1 to 10 with 50,000 replicates for burn-in and 50,000 replicates during analysis. The consensus number of subpopulations was determined based on the results of NJ tree analysis and the point where the posterior probability (LnP(D) began to plateau (Casa et al., 2008). Based on these, *k* = 4 was chosen as the optimal number of subpopulations. A graphical bar plot was then generated using the posterior membership coefficients. The NJ tree and STRUCTURE analyses were further confirmed by PCA conducted using R program. The analysis of molecular variance (AMOVA) for subpopulations and pairwise population differentiation (*F*_ST_) comparisons were performed in GenAIEx 6.501 software (Excoffier et al., 1992; Peakall et al., 1995; Peakall and Smouse, 2012). The genetic distances between the four identified subpopulations were calculated using POPGENE v 1.32 (Yeh et al., 1997) based on Nei’s (1978) unbiased genetic distance.

### Linkage Disequilibrium (LD) Analysis

Linkage disequilibrium was determined as the square value of correlation coefficient (*r*^2^) between all pairs of markers in TASSEL software version 3.0 using a sliding window (Bradbury et al., 2007). LD calculations were carried out within all 136 accessions and for each subpopulation identified using STRUCTURE. The LD extent was estimated separately for unlinked (loci on different chromosomes) and linked loci (on the same chromosome). All marker pairs with LD probability values of less than 0.05 were considered to be in significant LD. The significance of the *p*-values of *r*^2^ for each marker pair was performed using 10,000 permutations in TASSEL 3.0. The LD decay was determined when squared correlation coefficient, *r*^2^ = 0.02, and the scatterplots of estimated *r*^2^ values vs. distance (*c*M) between markers on a whole genome, chromosomes 1 and 2 were performed using curvilinear regression in SPSS software version 22.0 (IBM Corp., 2013). Genetic distances for 50 SSR markers for LD decay analysis were obtained from a genetic linkage map of sorghum constructed by Srinivas et al. (2009). The LD decay curves were fitted for the whole genome, chromosomes 1 and 2 using 50, 9 and 11 SSR markers, respectively.

### Association Analysis

The marker-trait associations were performed using GLM and MLM (Yu et al., 2006) in TASSEL 3.0 (Bradbury et al., 2007). In the GLM, the Q-matrix was integrated as a covariate in order to correct for the effects of population structure while Q and K matrices were both used in the MLM to correct for population structure and familial relatedness, respectively. The Q and K matrices were calculated using STRUCTURE 2.3.4 (Falush et al., 2012) and SPAGeDi (Hardy and Vekemans, 2002), respectively. All kinship values between individuals that were negative were set to zero. Significance of marker-trait associations was based on the threshold of *p* ≤ 1 × 10^-3^, a stringent Bonferroni correction determined by dividing 0.05 by 50 (number of markers used in this study) as described by Murray et al. (2009).

## Results

### Phenotypic Response to Early-Season Cold Stress

The summary statistics of phenotypic responses of genotypes to cold temperature stress is presented in **Table 1**. All traits were significantly different among genotypes both under cold stress and normal temperature regimes (Supplementary Table 3). Seedling emergence under cold stress ranged from 3.67 to 63.7% with a mean of 32.3% which is less than half of 67.9% recorded under normal temperature showing that sorghum remains sensitive to low temperature even in genotypes adapted to temperate climate (**Table 1**). Similarly, cold temperature treatment significantly reduced seedling vigor with mean seedling vigor under stress ranging from 1.15 to 5.00 compared to 1 to 3.92 under normal temperature. The overall mean seedling vigor score was 3.3 and 1.7 under cold and normal temperature treatments, respectively. Two other seedling growth parameters, seedling height and seedling dry matter, were also significantly different between genotypes and both were markedly affected by cold temperature treatment. Overall, mean seedling height was 18.84 cm under cold temperature compared to 45.21 cm under normal temperature with the range seemingly narrower under cold temperature (13.38–34.90 cm) than under normal temperature (25.17–63.77 cm). Seedling dry weight was also significantly different between genotypes under both temperature regimes with the highest dry matter score under cold temperature treatment (0.82 g) being smaller than the lowest score under normal temperature (0.89 g). Mean seedling dry weight for the cold and normal temperature regimes was 0.30 and 2.04 g, respectively.

**Table 1:** Summary of phenotypic data of all traits measured under early and normal planting at Kansas State University Agronomy research farm near Manhattan, KS, USA in 2010 and 2011.

Temperature regimes	Trait	Mean	Range	*SD*	*H*	^†^Accessions superior or comparable to SQR
Cold temperature	Seedling emergence (%)	32.3	3.67–63.67	14.52	0.58	19
	Seedling vigor (1–5)	3.30	1.15–5.00	0.72	0.71	11
	Seedling height (cm)	18.84	13.38–34.90	6.23	0.58	23
	Seedling dry weight (g)	0.30	0.01–0.82	0.59	0.53	41

Normal temperature	Seedling emergence (%)	67.55	46.00–92.58	10.60	0.36	29
	Seedling vigor (1–5)	1.70	1.00–3.92	0.58	0.41	25
	Seedling height (cm)	45.21	25.17–63.77	6.23	0.73	15
	Seedling dry weight (g)	2.04	0.89–3.64	0.57	0.56	31

*Seedling vigor rated as 1 = excellent vigor and 5 = poor vigor; SD, standard deviation; H, heritability estimates; ^†^Number of accessions significantly better or equal to SQR (cold-tolerant line).*

Given that the genotypes included in the study were adapted to cold weather condition, quite large number of them was comparable or superior to the known cold tolerance check. Overall 19, 11, 23, and 41 accessions were superior to or as good as SQR with respect to seedling emergence, vigor, seedling height and seedling dry weight, respectively, when grown under cold temperature (**Table 1**). Under normal planting, the proportion of genotypes superior to the check for seedling emergence and seedling vigor was higher, while it was lower for seedling height and about the same for seedling dry weight. Though the scores for all the traits were significantly affected by temperature regime, the pattern of genotypic response seems to be similar such that there was significant positive correlation between the temperature regimes for all of the traits. Accordingly, seedling emergence under cold and normal temperature were significantly correlated (*r* = 0.57). Similarly, correlation coefficients of *r* = 0.49, *r* = 0.39, and *r* = 0.54 were obtained for seedling vigor, seedling height and seedling dry weight, respectively (Supplementary Table 4). Broad-sense heritability (*H*^2^) for different parameters was estimated independently for the cold stress and normal temperature regimes. Heritability for the major cold temperature response traits such as emergence and seedling vigor, was high under cold temperature treatment, 0.58 and 0.71, respectively, compared to under normal temperature where *H*^2^ estimates were 0.36 and 0.41, respectively. Heritability for the other traits was comparable for both temperature treatments except seedling height was higher under normal temperature (**Table 1**). Moreover, all traits were highly correlated with each other except seedling emergence and seedling height under normal planting in 2010 and seedling emergence was not correlated with seedling height and seedling dry weight in 2011 (Supplementary Table 5)

### Genetic Diversity and Population Structure Analysis

Of the total of 67 markers used to genotype the materials, 50 of them showed sufficient polymorphism and hence included in the analysis. A total of 507 alleles were resolved across the 136 genotypes with an average of 10.14 alleles per locus. The average gene diversity was 0.84, ranging from 0.57 to 0.94 (**Table 2**). PIC values for the markers ranged from 0.49 to 0.94 with an average of 0.82.

**Table 2:** Genetic diversity of 136 sorghum accessions assessed by 50 SSR markers.

Statistics (per locus)	*Na*	*He*	*PIC*
Mean	10.14	0.84	0.82
Range	4–21	0.57–0.94	0.49–0.94
Stdev	3.38	0.06	0.07

*N_*a*_, observed number of alleles/locus; *He*, gene diversity (expected heterozygosity); *PIC*, polymorphism information content.*

**FIGURE 1:**
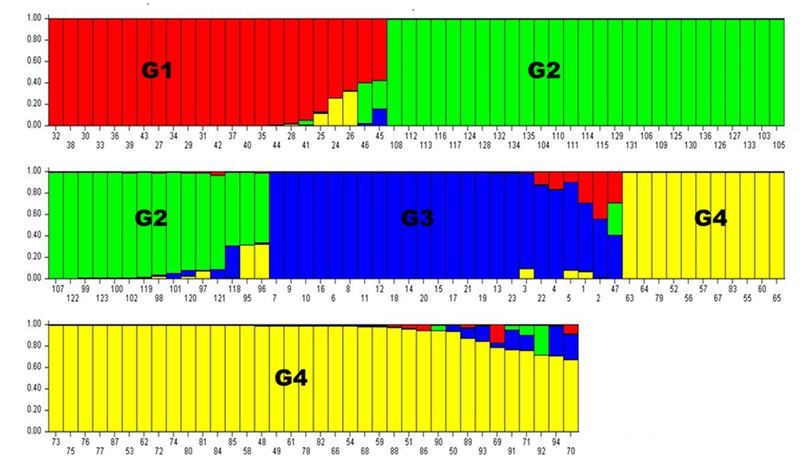
**Population structure analysis results.** Numbers on the *y*-axis show subpopulation memberships and the numbers on the *x*-axis show the accession numbers. The colors of the barplot indicate the four subpopulations identified by STRUCTURE program (G1 = *red*, G2 = *green*, G3 = *blue*, and G4 = Yellow.

Population structure analysis was performed using the STRUCTURE software version 2.3.4 (Falush et al., 2012). Based on the 0.8 as cutoff level for membership coefficient, the STRUCTURE analysis grouped the genotypes into four different subgroups, G1, G2, G3, and G4 consisting, 19, 39, 21, and 41 genotypes, respectively. The remaining 16 genotypes (about 12% of the population) had membership coefficients of <0.8 and hence were considered admixtures (**Figure 1**). Since the majority of the accessions were from Asia, geographic origin cannot be implicated as the cause for the subgroup pattern revealed by STRUCTURE analysis. However, the Asia region where the genotypes come from is vast covering Korea, Japan, China and Former Soviet Union. Geographical coordinates were not available to scrutinize the geography within the region. But the fact that genotypes from other regions were also scattered within these groups makes it clear that geographic origin has little role in affecting population structure. The genetic diversity statistics among the four subgroups also varied (**Table 3**). Group two (G2) had the highest total number of alleles (381) detected by the markers with average number of alleles per locus of 7.6, gene diversity 0.80 and PIC 0.77. G3 had the lowest total number of alleles (269) with an average of 5.4 alleles per locus.

**Table 3:** Summary statistics for subpopulations as identified by STRUCTURE analysis based on 50 SSR markers.

Subpopulation	Sample size	*NA*	*Na*	*He*	*PIC*
G1	23	280	5.6	0.70	0.66
G2	42	381	7.6	0.80	0.77
G3	24	269	5.4	0.71	0.67
G4	47	337	6.7	0.76	0.73

*NA, total number of alleles; *Na*, observed number of alleles per locus; *He*, gene diversity (expected heterozygosity); *PIC*, polymorphism information content.*

**FIGURE 2:**
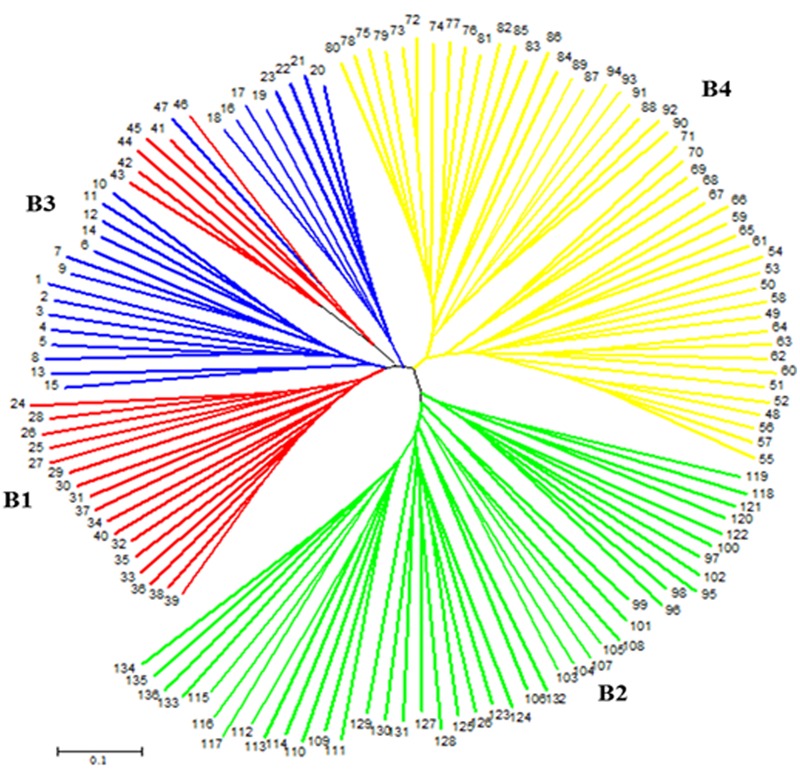
**An unrooted neighbor-joining tree (NJ) showing genetic relationships of 136 sorghum accessions based on 50 simple sequence repeat (SSR) markers.** The four subpopulations are labeled B1–B4 and numbers at tip of the branches indicate accession numbers. The color codes are from the structure output presented in **Figure 1**.

On the other hand, a NJ tree analysis conducted using PowerMarker clustered the genotypes into four major branches (B) (**Figure 2**). Four of the branches closely agree with the result from STRUCTURE analysis. About 74% of members of G1 and 100% of members of G2, G3 and G4 from the STRUCTURE analysis agree with members of B1, B2, B3, and B4 in NJ tree analysis. The association between the results of the STRUCTURE and NJ tree analysis was shown by the color-coded graphs as presented in **Figures 1, 2**.

### Principal Component Analysis

**FIGURE 3:**
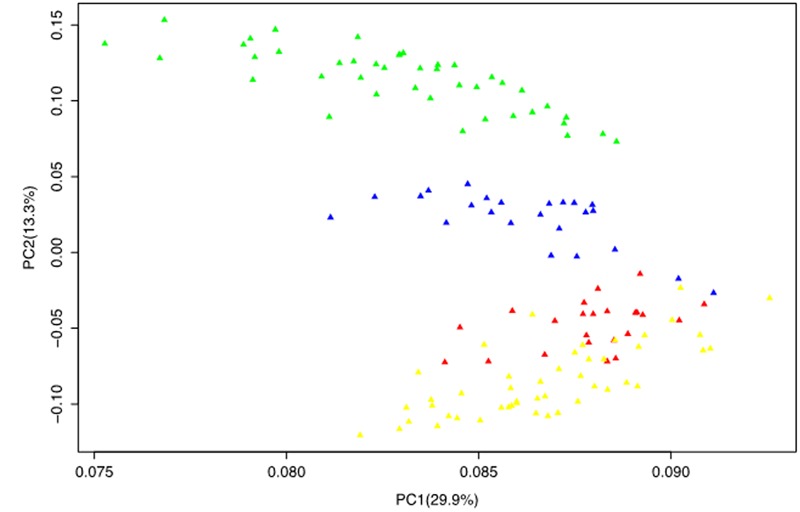
**Principal component analysis results based on genotypic data of 50 SSR markers.** The color codes are the same as those presented in the STRUCTURE (**Figure 1**) and NJ tree (**Figure 2**).

The PCA reduced the complex data set into principal components (axis) with the first two principal components jointly accounting for 43.2% of the total variation. Similar to the STRUCTURE and NJ tree, the PCA also grouped the population into four subgroups one of which clearly consisted of mixed genotypes that corresponds to the G1 subgroup of the STRUCTURE analysis and the B1 branch of the NJ tree. This was evident from the PCA plot of the first two principal components where the different groups were clearly disaggregated (**Figure 3**).

The AMOVA showed that 11 and 89% of total variation in the population was attributed to variation among and within subgroups, respectively (**Table 4**). Overall the population differentiation estimate (*F*_ST_) among the subgroups was 0.11 (data not shown). The pairwise *F*_ST_ estimates between subgroups showed G1 and G3 as closest with *F*_ST_ = 0.114, while the highest pairwise distance observed between G1 and G3 (*F*_ST_ = 0.146). A similar comparison based on Nei’s unbiased genetic distance for the four subgroups agree with the *F*_ST_ obtained through AMOVA.

**Table 4:** Analysis of molecular variance (AMOVA) for 136 sorghum accessions among and within four subpopulations as identified by STRUCTURE.

Source of variation	*df*	Sum of squares	Mean squares
Among populations	3	597.718	199.239^∗∗^
Within population	132	5131.061	38.872^∗∗^
Total	135	5728.779	39.1

*^∗∗^Significant at *P* ≤ 0.001; *df*, degrees of freedom.*

The cold tolerant genotypes appear to be randomly distributed across the population subgroups. Out of 19 genotypes that had superior or comparable emergence rate to SQR under cold temperature, 2, 6, 5, and 6 were in G1, G2, G3, and G4 subgroups, respectively. The distribution was similar for all other traits indicating that cold tolerance was not restricted to any subgroup in the population. However, on group mean basis, G4 appears to have desired scores for all parameters measured under cold temperature regime. Mean seedling emergence (38.8%), seedling vigor (3.3) and seedling dry matter (0.33 g) were highest in G4. The lowest emergence rate, seedling vigor and seedling height were recorded in G2 while the lowest seedling dry weight was in G1 (**Table 5**).

**Table 5:** Phenotypic responses of the accessions to temperature regimes across subpopulations detected by structure analysis based on 50 SSR markers.

			Mean^†^		Range		Accessions superior or comparable to SQR	
Traits	Sub- population	Sample size	Early planting	Normal planting	Early planting	Normal planting	Early planting	Normal planting
Emergence (%)	G1	23	35.10 (±18.90)	68.7 (±8.69)	5.3–62.7	46.4–80.8	2	3
	G2	42	30.40 (±13.30)	65.5 (±10.90)	9.3–55.7	46.0–83.8	6	8
	G3	24	32.30 (±12.80)	69.2 (±11.89)	3.7–55.3	49.3–92.6	5	9
	G4	47	36.80 (±16.30)	68.8 (±10.11)	12.0–63.7	46.0–90.3	6	9
	Overall	136	32.30 (±14.60)	67.9 (±10.60)	3.7–63.7	46.0–92.6	19	29

Seedling	G1	23	3.60 (±1.10)	1.73 (±0.66)	1.9–5.0	1.0–3.9	1	4
vigor (1–5)	G2	42	3.60 (±0.90)	1.92 (±0.79)	1.6–4.9	1.0–5.0	6	8
	G3	24	3.50 (±0.70)	1.67 (±0.56)	2.2–4.8	1.0–3.3	1	5
	G4	47	3.30 (±0.70)	1.68 (±0.51)	2.3–5.0	1.0–3.7	3	8
	Overall	136	3.30 (±0.80)	1.70 (±0.58)	1.2–5.0	1.0–5.0	11	25

Seedling	G1	23	19.22 (±3.64)	45.71 (±5.12)	13.8–26.0	35.0–56.4	5	2
height (cm)	G2	42	18.74 (±3.06)	44.31 (±5.56)	13.4–24.9	36.0–58.1	8	3
	G3	24	19.10 (±3.53)	45.05 (±6.82)	15.0–34.9	25.2–56.6	5	3
	G4	47	19.36 (±2.67)	45.80 (±6.82)	15.1–26.0	33.6–63.8	5	7
	Overall	136	18.84 (±6.23)	45.21 (±6.23)	13.4–34.9	25.2–63.8	23	15

Seedling dry	G1	23	0.26 (±0.09)	2.04 (±0.47)	0.1–0.4	0.9–3.0	4	4
weight (g)	G2	42	0.29 (±0.09)	1.94 (±0.61)	0.1–0.5	0.9–3.6	10	8
	G3	24	0.31 (±0.13)	2.14 (±0.52)	0.0–0.8	1.1–3.1	10	8
	G4	47	0.33 (±0.08)	2.07 (±0.59)	0.2–0.5	0.9–3.6	17	11
	Overall	136	0.30 (±0.59)	2.04 (±0.57)	0.0–0.8	0.9–3.6	41	31

*SQR, shan qui red; ^†^Numbers in parentheses are standard deviations.*

### Linkage Disequilibrium (LD) Analysis

**FIGURE 4:**
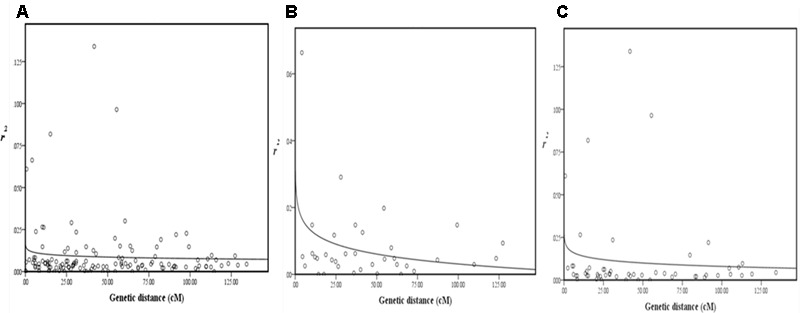
**Scatterplots of estimates of *r*^2^ of SSR marker pairs for (A)** whole genome, **(B)** chromosome 1, and **(C)** chromosome 2, showing LD decays as measured by *r*^2^ against genetic distance (*c*M). The decay curves were plotted with log. (*r*^2^) values using curvilinear regression in SPSS software.

Pairwise LD estimates were performed on the entire population and on the subgroups using all 50 marker loci. On the entire panel, the average coefficient of determination, *r*^2^, of marker locus pairs was 0.016, and 13.71% of the total possible marker locus pairs were in significant LD (*p* < 0.05) (**Table 6**). The average *r*^2^ of the linked (intra-chromosomal) and unlinked (inter-chromosomal) marker locus pairs were 0.016 and 0.014, respectively, with 13.75 and 13.38% in significant LD (*p* < 0.05), in that order. The percentage of linked marker locus pairs in significant LD (*p* < 0.05) was higher than that of unlinked marker locus pairs. For the overall marker pairs, the mean *r*^2^ in the subgroups ranged from 0.031 in G2 to 0.060 in G1. The percentage of pairwise marker locus pairs in significant LD in the four subgroups ranged from 5.06 in G2 to 10.29% in G4. Furthermore, the average *r*^2^ between marker locus pairs for the four subgroups ranged from 0.030 to 0.062 for the linked markers and 0.032 to 0.061 for the unlinked markers which is higher than that of the entire panel. The percentage of marker locus pairs in significant LD in the four subgroups ranged from 3.01 to 9.30 for the linked markers and from 4.65 to 9.25 for the unlinked markers. For the whole genome, the LD decayed at less than 0.5*c*M was estimated, while for chromosomes 1 and 2, LD decayed at less than 3 and 5*c*M, respectively (**Figure 4**).

**Table 6:** Whole genome level LD in the entire panel and the subpopulations obtained from the structure output.

Subpopulation Groups	^†^Overall		^†^Linked		^§^Unlinked	
	*r*^2^	£Significant LD (%)	*r*^2^	£Significant LD (%)	*r*^2^	£Significant LD (%)
G1	0.060	4.98	0.051	3.01	0.061	4.65
G2	0.031	5.06	0.030	7.38	0.032	4.81
G3	0.059	5.80	0.062	4.72	0.058	6.01
G4	0.037	10.29	0.031	9.30	0.037	9.25
Total	0.016	13.71	0.016	13.75	0.014	13.38

*^†^The whole set of marker pairs (linked and unlinked marker pairs). ^‡^Pairs of markers on the same chromosome. ^§^Pairs of markers from different chromosomes. £Significant threshold, *p* < 0.05, which determine whether pairwise LD estimate is statistically significant.*

### Association Analysis

The results for marker-trait association are presented in **Table 7**. Both GLM and MLM were used to find significant association between markers and all seedling parameters collected under both temperature regimes. Under cold temperature treatment, 6 SSR markers were found to be significantly (*p* < 0.05) associated with the seedling traits each contributing 10.8–24.8% of the phenotypic variation observed. Using GLM, two SSR markers, *Xtxp*34 on chromosome 3 and *Xtxp*319 on chromosome 1 were found to be significantly associated with seedling emergence under cold temperature in 2010 explaining 24.2 and 10.8% of phenotypic variation, respectively. But both of these markers were not significant with the MLM procedure. However, in 2011 and across the two seasons, *Xtxp*88 also located on chromosome 1 was found to be significantly associated with seedling emergence both in GLM and MLM procedures explaining 11.2 and 10.9% of phenotypic variation with GLM and 12.1 and 11.9 with MLM, respectively. For seedling dry weight, *Xtxp*304 and *Xtxp*211, located on chromosomes 1 and 2, respectively, were significantly associated with the trait for the 2011 testing each explaining 22.6% of phenotypic variation. There was no marker significantly associated with seedling height for independent environment analysis but in the combined data, *Xtxp*20 was found to be significantly associated with the trait explaining 24.8% of phenotypic variation for seedling height. Under normal temperature, *Xtxp*319 in 2010 and *Xtxp7* in 2011 seasons were found to be significantly associated with seedling emergence using GLM explaining 13.3 and 21% phenotypic variations, respectively. However, no significant marker-trait associations were observed in both environments using MLM. Again, *Xtxp*319 was significantly associated with seedling dry weight across environments accounting for 12.9 and 11.7% phenotypic variations in GLM and MLM models, respectively. Among the markers tested in this study, *Xtxp*88 showed a strong and consistent marker-trait association both in GLM and MLM models.

**Table 7:** Significant marker-trait associations identified under early and normal planting regimes using both general linear model (GLM) and MLM under two environments and combined across environments.

Planting regime	Environment	Trait	Markers	Chr.	Marker position (*c*M)	*p*-value (*Q*)	*r*^2^ (*%*)	*p*-value (*Q*+*K*)	*r*^2^ (*%*)
Early planting	2010	Seedling emergence	Xtxp34	3	97.2	7.39 × 10^-4^	24.2	8.57 × 10^-2^	14.5
			Xtxp319	1	11.5	3.37 × 10^-4^	10.8	3.18 × 10^-2^	4.3
	2011	Seedling emergence	**Xtxp88**	1	66.3	2.82 × 10^-4^	11.2	3.81 × 10^-4^	12.1
		Seedling dry weight	Xtxp304	2	40.4	1.51 × 10^-4^	22.6	9.60 × 10^-3^	17.6
			Xtxp211	2	32.2	1.52 × 10^-4^	22.6	14.7 × 10^-2^	10.4
	Across years	Seedling emergence	**Xtxp88**	1	66.3	4.67 × 10^-4^	10.9	5.01 × 10^-4^	11.9
		Seedling height (cm)	Xtxp20	10	20.9	6.09 × 10^-5^	24.8	3.50 × 10^-3^	20.6

Normal planting	2010	Seedling emergence	Xtxp319	1	11.5	3.36 × 10^-5^	13.3	1.18 × 10^-3^	8.8
	2011	Seedling emergence	Xtxp7	2	116.1	9.38 × 10^-4^	21	4.62 × 10^-3^	21
	Across years	Seedling dry weight	**Xtxp319**	1	11.5	1.63 × 10^-4^	12.9	8.64 × 10^-4^	11.7

*Markers in bold are *p-*values ≤ 1 × 10^*–3*^, the Bonferroni-corrected threshold probability at 5% significance level; Chr., chromosome; *c*M, centiMorgan.*

## Discussion

The sensitivity of sorghum to cool temperatures especially during establishment and early growth stages cannot be overemphasized. Temperatures below 15°C can reduce seed germination, seedling emergence and vigor limiting production both temporally and spatially. Reduced seedling vigor caused by low temperature may not necessarily lead to reduced yield if the stress is removed (temperature warms up) at later stages (Maulana and Tesso, 2013). But low germination and poor emergence under cold stress can result in poor stand establishment that may directly translate to reduced yield. While this is the case for most summer crops, sorghum is particularly sensitive to cold temperature given its inherent adaptation to warm and dry weather. As a result, sorghum planting in temperate environments is often delayed by 3–4 weeks after maize until late spring or early summer for soil temperature to warm up which requires that early maturing cultivars be planted to make sure that the crop matures before freezing temperatures hit in the fall. The problem is even more critical in northern latitudes where the time window becomes even narrower requiring that extra early varieties be grown. Any technology that permits field planting early in the spring can extend the growing period that may allow production of full season hybrids for improved yield. Hence developing and deploying cold tolerant sorghum hybrids has enormous impact on increasing production both through increasing acreage by expanding production to areas traditionally considered too cold for the crop and through increasing productivity through deployment of high yield full season hybrids in acres currently allocated to the crop.

Despite its origin in tropical climate, sorghum displays significant genetic variability for cold tolerance. For this study we emphasized on screening sorghum accessions collected from cooler regions of the world mainly from China, Korea, Japan, and the former Soviet Union in order to identify potential sources of cold tolerance for use in breeding programs. The specific interest on sorghums of these regions was based on previous observations that sorghums from colder places like China have consistently exhibited improved cold tolerance than those from other regions (Franks et al., 2006). We have also attempted to determine the population structure among the accessions to see the pattern of distribution of the tolerant sources to guide future research and germplasm choice.

Field screening of the accessions showed significant variability for response to cold stress with many of them showing superior or comparable level of tolerance with the cold tolerant check SQR. Given that the accessions were purposely sampled from colder regions, the result was not surprising. It also shows that sources of improved germination and seedling vigor under cold stress are abundant in sorghum. Out of 136 accessions evaluated, 19 were superior to SQR with respect to emergence and 11 for seedling vigor. Many accessions also had improved seedling traits such as seedling height and seedling biomass compared to SQR.

Past researches have reported significant association between early season cold tolerance and concentration of tannin in the testa layer of sorghum grain with QTLs affecting both traits essentially overlapping (Knoll et al., 2008; Wu et al., 2012). While tannin is considered among anti-oxidant molecules desired for their health benefits, tannin undermines the conventional uses of sorghum as animal feed and human food. Future efforts to enhance cold tolerances for these traditional uses should either dissect the genetic mechanisms for tannin accumulation and introduce variants of alleles with defective functions or focus on tropical sorghum germplasm for new sources of cold tolerance.

Similar to the phenotypic variation (data not shown) and the impressive difference for cold tolerance traits, the accessions also expressed significant genetic variability with very high average number of alleles per locus of 10.14 (**Table 3**) compared to 9 (Ramu et al., 2013) and 5.6 (Folkertsma et al., 2005) previously reported in grain sorghum accessions. Given that the accessions were largely derived from one continent (Asia), the level of genetic variation observed was very high and confirms the tremendous genetic variation harbored in this species. The STRUCTURE analysis (**Figure 1**) conducted on the marker distance matrix grouped the accessions into four subgroups. In contrast to the previous studies that sorted accessions based on geographic origins (Wang et al., 2013), the pattern of subgrouping in the current study does not seem to reflect geographic adaptation. Almost all of the four population subgroups contained accessions from two or more countries and accessions from the same country were also present in all of the four subgroups. Nevertheless, few other previous studies grouped sorghum based on racial category (Morris et al., 2013). Since the accessions used in the current study were not characterized for racial groups, it was not possible to relate the observed genetic differences with races. But from the field observation of the accessions races bicolor and guinea were obviously absent though intermediate groups of other races may be present.

The NJ tree analysis also revealed similar result by grouping the accessions into four distinct subgroups (**Figure 2**) which corresponds to outputs generated by the STRUCTURE analysis. In addition to the number of subgroups, individuals within subgroups developed by both NJ tree and STRUCTURE analyses were very consistent except minor changes. This can be revealed from the color coded display of both the STRUCTURE and NJ tree outputs (**Figures 1, 2**). Results of both analyses indicate though the variation among the accessions was broad, majority of accessions studied were somehow broadly distributed in the region to the extent that the effect of geographic segregation became invisible. Two likely scenarios may explain the current results: Firstly, the accessions may be introductions to the Asian continent perhaps from countries in Africa rather than evolved there and hence may share pedigree from a possible common source country in Africa. Secondly, germplasm movement between countries in Asia may be lax at some point that facilitated free movement of germplasm and cultivars between countries in the continent leading to most of the sampled accessions having common source. The fact that the high tannin trait was shared among the most promising cold tolerant accessions regardless of the country where they come from supports this argument.

Although the number of markers used in this study was not large enough to offer high resolution map, we thought highlighting on marker-trait association, though may be crude, may serve as basis for future studies on the problem and to see if mapping results from bi-parental population can be reproduced. Accordingly, GLM based association mapping of seedling emergence for both 2010 and 2011 data showed that *Xtxp*34 and *Xtxp*319 were significantly associated with seedling emergence in 2010 and *Xtxp*88 in 2011. Also *Xtxp*304 and *Xtxp*211 were associated with seedling dry weight in 2011. Across locations, *Xtxp*88 came out as the most significant marker associated with seedling emergence. No marker was significant for seedling dry weight in the combined data. Both *Xtxp*319 and *Xtxp*88 were located on chromosome 1 in sorghum and were reported to be associated with early-season cold tolerance in previous studies conducted on a recombinant inbred line (RIL) population derived from African cold sensitive line SRN39 and Chinese cold tolerant genotype SQR (Knoll et al., 2008). *Xtxp*211 located on chromosome 2 was also reported to be associated with early-season cold tolerance in a different RIL population that involved Tx430 and another cold tolerant Chinese material PI610727 (Burrow et al., 2011). *Xtxp*304 has not been reported in previous studies but it was found only 8*c*M away from *Xtxp*211 on chromosome 2. Under normal planting *Xtxp*319 located on chromosome 1 and *Xtxp*7 located on chromosome 2 (not reported in previous studies) were significantly associated with seedling emergence and *Xtxp*319 with seedling dry weight. The significant association between marker *Xtxp*319 and seedling dry weight may be the result of its association with seedling emergence which is perhaps a prerequisite for seedling growth and vigor.

## Conclusion

There is tremendous genetic variability for early-season cold tolerance among sorghum accessions from cooler regions of the world. The cold tolerance sources identified in this study were clustered across wide sorghum geographies indicating that alleles associated with tolerance to the stress may vary in different genetic backgrounds. Therefore, besides those from cooler regions of the world, future efforts to identify new sources of cold tolerance should consider evaluation and screening of germplasm from diverse geographic regions. The bleach test results indicate that cold tolerance continues to be associated with high tannin in kaoliang sorghums and its derivatives. Therefore, future search for tannin-free sources of cold tolerance should focus on tropical highlands. However, cold tolerance sources should be checked for tannin levels before including into breeding programs. Molecular markers poise as powerful tools for enhancing breeding efficiency for cold tolerance. This study identified six SSR markers associated with early-season cold tolerance traits using association mapping approach. Some of the markers were detected on chromosomal regions previously reported to harbor QTLs associated with early-season cold tolerance in sorghum. Once validated they may serve as tools in marker-assisted breeding and to screen larger pool of genotypes to identify additional sources of cold tolerance.

## Author Contributions

FM is the primary author of this manuscript. He was in charge of the conduct of the experiments under both cold temperature stress and normal temperature conditions in both test seasons as well as analysis of the data and drafting of the manuscript. DW actively participated in the preparation of this manuscript especially through assisting with statistical analysis of the data and review of the draft manuscript. TT is the corresponding author on this manuscript. He is the PI of the project under which this study was carried out and was responsible for supervising the conduct of the experiment, analysis of the data and for finalizing the review and submission of the manuscript.

## Conflict of Interest Statement

The authors declare that the research was conducted in the absence of any commercial or financial relationships that could be construed as a potential conflict of interest.

## Abbreviations

LD: linkage disequilibrium
MLM: mixed linear model
PCA: principal component analysis
PCR: polymerase chain reaction
PIC: polymorphic information content
QTL: quantitative trait loci
SQR: Shan qui red
