# Supplementary material for: Sorghum Landrace Collections from Cooler Regions of the World Exhibit Magnificent Genetic Differentiation and Early Season Cold Tolerance
Source: Front Plant Sci. 2017 May 9;8:756. doi: 10.3389/fpls.2017.00756 (PMC5422509; doi:10.3389/fpls.2017.00756)
Supplement: Supplementary file 1 [file Data_Sheet_1.docx]

**Supplementary Tables**

**Table 1** List of 136 sorghum accessions used in this study and their subpopulation membership coefficients (Q-matrix)

| No. | PI No. | Identifier | Collection site/origin | Q1 | Q2 | Q3 | Q4 |
| --- | --- | --- | --- | --- | --- | --- | --- |
| 1 | PI563634 | LR 431-1 | China | 0.289 | 0.001 | 0.643 | 0.067 |
| 2 | PI92264 | IS12744 | China | 0.438 | 0.001 | 0.55 | 0.012 |
| 3 | - | BTx378 | United States | 0.01 | 0.002 | 0.894 | 0.094 |
| 4 | PI609465 | SC 1345 | United States | 0.163 | 0.002 | 0.83 | 0.005 |
| 5 | PI30204 | Japanese dwarf broomcorn | United States | 0.094 | 0.001 | 0.823 | 0.083 |
| 6 | PI567911 | Bai Nian Gao Liang (Jin Xi) | China | 0.002 | 0.001 | 0.997 | 0.001 |
| 7 | PI82335 | Kaoliang-Wx | Korea | 0.001 | 0.001 | 0.998 | 0.001 |
| 8 | PI563703 | LR 2490-3 | China | 0.002 | 0.001 | 0.997 | 0.001 |
| 9 | PI607408 | PI 607408 | China | 0.001 | 0.001 | 0.998 | 0.001 |
| 10 | PI547919 | Bai Li Gao Liang | China | 0.001 | 0.001 | 0.998 | 0.001 |
| 11 | PI234456 | PI 234456 | Japan | 0.001 | 0.001 | 0.997 | 0.001 |
| 12 | PI92268 | PI 92268 | China | 0.001 | 0.001 | 0.997 | 0.001 |
| 13 | PI563699 | LR 2483-1 | China | 0.007 | 0.003 | 0.988 | 0.002 |
| 14 | PI92261 | IS 12741 | China | 0.001 | 0.001 | 0.996 | 0.001 |
| 15 | PI455541 | ETS 3633 | Ethiopia | 0.003 | 0.001 | 0.995 | 0.001 |
| 16 | PI563692 | LR 2470-1 | China | 0.001 | 0.001 | 0.998 | 0.001 |
| 17 | PI267113 | K-892 | Africa | 0.003 | 0.001 | 0.995 | 0.001 |

| No. | PI No. | Identifier | Collection site/origin | Q1 | Q2 | Q3 | Q4 |
| --- | --- | --- | --- | --- | --- | --- | --- |
| 18 | PI55123 | Hemaise | Sudan | 0.001 | 0.001 | 0.997 | 0.001 |
| 19 | PI550666 | PI 550666 | Former Soviet Union | 0.002 | 0.004 | 0.992 | 0.003 |
| 20 | PI76409 | She-jen (snake eye) | China | 0.001 | 0.001 | 0.996 | 0.002 |
| 21 | PI542739 | Ping Ding Xiang | China | 0.002 | 0.002 | 0.995 | 0.001 |
| 22 | PI23231 | Brown Kaoliang | China | 0.12 | 0.006 | 0.872 | 0.002 |
| 23 | PI267105 | K-11 | Former Soviet Union | 0.002 | 0.006 | 0.986 | 0.007 |
| 24 | PI563943 | PI 563943 | China | 0.736 | 0.001 | 0.002 | 0.262 |
| 25 | PI550843 | PI 550843 | Italy | 0.869 | 0.001 | 0.011 | 0.119 |
| 26 | PI585378 | IS 24692 | India | 0.67 | 0.002 | 0.004 | 0.324 |
| 27 | PI656027 | SRN39 | Sudan | 0.996 | 0.001 | 0.001 | 0.001 |
| 28 | PI563639 | LR 432-1 | China | 0.974 | 0.001 | 0.007 | 0.018 |
| 29 | PI92272 | IS 12750 | China | 0.995 | 0.001 | 0.003 | 0.001 |
| 30 | PI246699 | IS 1024 | India | 0.997 | 0.001 | 0.001 | 0.001 |
| 31 | PI562755 | IS 2212 | United States | 0.995 | 0.001 | 0.002 | 0.002 |
| 32 | PI586454 | Leoti | Hungary | 0.998 | 0.001 | 0.001 | 0.001 |
| 33 | PI267115 | K-34 | Former Soviet Union | 0.997 | 0.001 | 0.001 | 0.001 |
| 34 | PI586524 | IS 27929 | China | 0.996 | 0.002 | 0.001 | 0.001 |

**Table 1** continued

**Table 1** continued

| No. | PI No. | Identifier | Collection site/origin | Q1 | Q2 | Q3 | Q4 |
| --- | --- | --- | --- | --- | --- | --- | --- |
| 35 | PI542718 | San chi San | China | 0.993 | 0.001 | 0.004 | 0.003 |
| 36 | PI87355 | Bomususu | Korea | 0.997 | 0.001 | 0.001 | 0.001 |
| 37 | PI563676 | LR 2417(a) | China | 0.994 | 0.002 | 0.003 | 0.001 |
| 38 | PI607404 | PI 607404 | China | 0.998 | 0.001 | 0.001 | 0.001 |
| 39 | PI607407 | PI 607407 | China | 0.997 | 0.001 | 0.001 | 0.001 |
| 40 | PI501620 | PI 501620 | Yemen | 0.994 | 0.002 | 0.003 | 0.001 |
| 41 | PI563702 | LR 2490-2 | China | 0.949 | 0.037 | 0.01 | 0.005 |
| 42 | PI92270 | MN 2740 | China | 0.995 | 0.001 | 0.003 | 0.002 |
| 43 | - | Gao Gaoliang | China | 0.997 | 0.001 | 0.002 | 0.001 |
| 44 | PI90769 | Red Kaoliang | China | 0.992 | 0.001 | 0.006 | 0.001 |
| 45 | PI563700 | LR 2483-2 | China | 0.578 | 0.262 | 0.158 | 0.001 |
| 46 | PI607931 | Tx2911 | United States | 0.6 | 0.376 | 0.021 | 0.002 |
| 47 | PI655993 | Tx399 | United States | 0.289 | 0.297 | 0.413 | 0.001 |
| 48 | PI76409 | North West Gold Kaoliang | China | 0.007 | 0.001 | 0.001 | 0.992 |
| 49 | PI550859 | PI550859 | Italy | 0.004 | 0.001 | 0.003 | 0.992 |
| 50 | PI511832 | Big Yellow Umbrella | China | 0.003 | 0.001 | 0.06 | 0.935 |
| 51 | PI267112 | K-819 | Former Soviet Union | 0.03 | 0.001 | 0.009 | 0.96 |

**Table 1** continued

| No. | PI No. | Identifier | Collection site/origin | Q1 | Q2 | Q3 | Q4 |
| --- | --- | --- | --- | --- | --- | --- | --- |
| 52 | PI586526 | IS 27931 | China | 0.001 | 0.001 | 0.001 | 0.997 |
| 53 | PI574605 | FS73015-D001 | United States | 0.002 | 0.001 | 0.002 | 0.995 |
| 54 | PI563656 | L 1791B | China | 0.003 | 0.001 | 0.008 | 0.987 |
| 55 | PI63923 | White Kaoliang | China | 0.002 | 0.001 | 0.001 | 0.996 |
| 56 | PI567795 | Danyang Local | South Korea | 0.001 | 0.001 | 0.001 | 0.997 |
| 57 | PI656025 | Shan qui red | China | 0.002 | 0.001 | 0.001 | 0.997 |
| 58 | PI92267 | Dwarf Yellow Milo | China | 0.001 | 0.001 | 0.004 | 0.993 |
| 59 | PI563701 | LR 2490-1 | China | 0.003 | 0.001 | 0.016 | 0.979 |
| 60 | PI666147 | IS 4225 | China | 0.002 | 0.001 | 0.001 | 0.996 |
| 61 | - | VA110 | United States | 0.004 | 0.003 | 0.001 | 0.992 |
| 62 | PI550610 | Durra Belaya | Syria | 0.002 | 0.001 | 0.002 | 0.995 |
| 63 | PI607403 | PI 607403 | China | 0.001 | 0.001 | 0.001 | 0.998 |
| 64 | PI92263 | MN 2735 | China | 0.001 | 0.001 | 0.001 | 0.998 |
| 65 | PI563667 | L 1999B-13 | China | 0.001 | 0.001 | 0.002 | 0.996 |
| 66 | PI71309 | Lo leung mai | China | 0.002 | 0.003 | 0.006 | 0.989 |
| 67 | PI547915 | Bai Ruan Gao Liang | China | 0.001 | 0.001 | 0.001 | 0.997 |
| 68 | PI88004 | Susu zairai shu | Korea | 0.003 | 0.011 | 0.002 | 0.984 |

**Table 1** continued

| No. | PI No. | Identifier | Collection site/origin | Q1 | Q2 | Q3 | Q4 |
| --- | --- | --- | --- | --- | --- | --- | --- |
| 69 | PI568047 | San Sui Jiao Zi | China | 0.169 | 0.002 | 0.043 | 0.786 |
| 70 | PI563576 | LV 129 | China | 0.084 | 0.003 | 0.236 | 0.677 |
| 71 | PI250230 | MN 4116 | Pakistan | 0.003 | 0.093 | 0.146 | 0.758 |
| 72 | PI567797 | Pyungchang Local | South Korea | 0.002 | 0.001 | 0.002 | 0.995 |
| 73 | PI547928 | Da Luo Chui | China | 0.001 | 0.001 | 0.001 | 0.996 |
| 74 | - | BTx3042 | United States | 0.001 | 0.001 | 0.002 | 0.995 |
| 75 | PI568044 | Bai Ri Hong | China | 0.001 | 0.001 | 0.002 | 0.996 |
| 76 | PI563668 | L 1999B-14 | China | 0.001 | 0.001 | 0.002 | 0.996 |
| 77 | PI563705 | LR2505 | China | 0.003 | 0.001 | 0.001 | 0.996 |
| 78 | PI92271 | IS 12749 | China | 0.005 | 0.002 | 0.003 | 0.991 |
| 79 | PI563686 | LR 2433 | China | 0.001 | 0.001 | 0.001 | 0.998 |
| 80 | PI266962 | IS 13238 | China | 0.003 | 0.001 | 0.001 | 0.995 |
| 81 | PI548014 | Huang Luo Mian | China | 0.003 | 0.001 | 0.002 | 0.995 |
| 82 | PI81216 | Migna | Yemen | 0.001 | 0.008 | 0.001 | 0.99 |
| 83 | PI90267 | Kaoliang | Korea | 0.002 | 0.001 | 0.001 | 0.997 |
| 84 | PI563638 | LR 431-2 | China | 0.001 | 0.002 | 0.001 | 0.995 |
| 85 | PI586532 | IS 27938 | China | 0.003 | 0.001 | 0.002 | 0.995 |

**Table 1** continued

| No. | PI No. | Identifier | Collection site/origin | Q1 | Q2 | Q3 | Q4 |
| --- | --- | --- | --- | --- | --- | --- | --- |
| 86 | PI267127 | K-24 | Former Soviet Union | 0.051 | 0.001 | 0.001 | 0.947 |
| 87 | PI547991 | Dao Zai Tou | China | 0.002 | 0.001 | 0.002 | 0.996 |
| 88 | PI601918 | MP 346 | United States | 0.016 | 0.007 | 0.005 | 0.972 |
| 89 | PI267130 | K-517 | Former Soviet Union | 0.026 | 0.006 | 0.093 | 0.876 |
| 90 | - | Tx436 | United States | 0.002 | 0.049 | 0.003 | 0.947 |
| 91 | PI563634 | LR 427 | China | 0.002 | 0.047 | 0.18 | 0.772 |
| 92 | - | BTx430 | United States | 0.002 | 0.279 | 0.001 | 0.718 |
| 93 | PI220636 | Nai-Shaker | Afghanistan | 0.004 | 0.006 | 0.147 | 0.843 |
| 94 | PI610730 | San Er Sui | China | 0.005 | 0.007 | 0.28 | 0.708 |
| 95 | PI607402 | PI 607402 | China | 0.001 | 0.679 | 0.001 | 0.318 |
| 96 | PI92260 | IS12740 | China | 0.009 | 0.65 | 0.014 | 0.327 |
| 97 | PI88000 | Mokutakususu | Korea | 0.013 | 0.905 | 0.006 | 0.076 |
| 98 | PI68003 | Kei ko she jen hing | China | 0.009 | 0.954 | 0.012 | 0.025 |
| 99 | PI585372 | IS24666 | Lebanon | 0.001 | 0.989 | 0.006 | 0.003 |
| 100 | PI610743 | Jiao Zi | China | 0.005 | 0.981 | 0.011 | 0.003 |
| 101 | PI655978 | Tx2737 | United States | 0.003 | 0.946 | 0.049 | 0.002 |
| 102 | PI455549 | ETS 3638 | Ethiopia | 0.011 | 0.98 | 0.002 | 0.007 |

**Table 1** continued

| No. | PI No. | Identifier | Collection site/origin | Q1 | Q2 | Q3 | Q4 |
| --- | --- | --- | --- | --- | --- | --- | --- |
| 103 | PI267129 | K-403 | China | 0.001 | 0.994 | 0.003 | 0.001 |
| 104 | PI562749 | IS 2033 | United States | 0.001 | 0.997 | 0.001 | 0.001 |
| 105 | PI567974 | Da Guan Dong | China | 0.002 | 0.994 | 0.002 | 0.001 |
| 106 | PI563726 | LR 2556-2 | China | 0.001 | 0.996 | 0.002 | 0.001 |
| 107 | PI607409 | PI 607409 | China | 0.002 | 0.994 | 0.001 | 0.003 |
| 108 | PI542764 | Jilin Hei Long Jiang-22 | China | 0.001 | 0.998 | 0.001 | 0.001 |
| 109 | PI562756 | IS 2216 | United States | 0.002 | 0.996 | 0.001 | 0.001 |
| 110 | PI656015 | Ajabsido | Sudan | 0.001 | 0.997 | 0.001 | 0.001 |
| 111 | PI565116 | SDS 1412 | Zimbabwe | 0.001 | 0.997 | 0.001 | 0.001 |
| 112 | PI563673 | LR2410 | China | 0.001 | 0.998 | 0.001 | 0.001 |
| 113 | PI586448 | Cody | Hungary | 0.001 | 0.998 | 0.001 | 0.001 |
| 114 | PI576434 | SC 1103 | United States | 0.001 | 0.997 | 0.001 | 0.001 |
| 115 | PI567929 | Da Qing Ye (Yang Qu) | China | 0.001 | 0.997 | 0.001 | 0.001 |
| 116 | PI563689 | LR 2462-2 | China | 0.001 | 0.998 | 0.001 | 0.001 |
| 117 | PI563402 | IS10497 | United States | 0.001 | 0.998 | 0.000 | 0.001 |
| 118 | PI567911 | Bai She Yan (Sui Zhong) | China | 0.003 | 0.685 | 0.299 | 0.013 |
| 119 | PI192876 | Katengu | Indonesia | 0.003 | 0.98 | 0.003 | 0.014 |

**Table 1** continued

| No. | PI No. | Identifier | Collection site/origin | Q1 | Q2 | Q3 | Q4 |
| --- | --- | --- | --- | --- | --- | --- | --- |
| 120 | PI586445 | Blackhull kafir | Hungary | 0.009 | 0.908 | 0.056 | 0.027 |
| 121 | - | R-45 | United States | 0.029 | 0.879 | 0.089 | 0.004 |
| 122 | PI267128 | K-385 | Former Soviet Union | 0.001 | 0.992 | 0.002 | 0.005 |
| 123 | PI563725 | LR 2556-1 | China | 0.007 | 0.985 | 0.001 | 0.007 |
| 124 | PI563666 | L 1999B-11 | China | 0.001 | 0.998 | 0.001 | 0.001 |
| 125 | PI563675 | LR 2412-2 | China | 0.001 | 0.996 | 0.001 | 0.002 |
| 126 | PI563727 | LR 2572 | China | 0.001 | 0.995 | 0.003 | 0.001 |
| 127 | PI563632 | LR 423 | China | 0.003 | 0.995 | 0.002 | 0.001 |
| 128 | PI267117 | K-540 | Former Soviet Union | 0.001 | 0.998 | 0.001 | 0.001 |
| 129 | PI563433 | IS 10731 | United States | 0.001 | 0.997 | 0.001 | 0.001 |
| 130 | PI563643 | L 1097B | China | 0.002 | 0.996 | 0.001 | 0.001 |
| 131 | PI563404 | IS 10505 | United States | 0.002 | 0.997 | 0.001 | 0.001 |
| 132 | PI267120 | K-47 | Former Soviet Union | 0.001 | 0.998 | 0.001 | 0.001 |
| 133 | PI562769 | Purdue 81659-2 | United States | 0.002 | 0.995 | 0.001 | 0.002 |
| 134 | PI563650 | L1603B | China | 0.001 | 0.998 | 0.001 | 0.001 |
| 135 | PI656058 | P9517 | United States | 0.001 | 0.998 | 0.001 | 0.001 |
| 136 | PI267126 | K-357 | Former Soviet Union | 0.001 | 0.996 | 0.002 | 0.001 |

**Table 2** List and characteristics of 50 SSR markers used for analysis of the cold tolerance sorghum germplasm population.

| Marker | LG | Type of SSR(s) | Forward primer (5'to 3') | Reverse primer (5' to 3') | Size(bp) | T_a_ |
| --- | --- | --- | --- | --- | --- | --- |
| Xtxp4 | 2 | (GA)23 | AATACTAGGTGTCAGGGCTGTG | ATGTAACCGCAACAACCAAG | 173 | 55 |
| Xtxp6 | 6 | (CT)33 | ATCGGATCCGTCAGATC | TCTAGGGAGGTTGCCAT | 120 | 50 |
| Xtxp7 | 2 | (CT)14 | ACATCTACTACCCTCTCACC | ACACATCGAGACCAGTTG | 200 | 50 |
| Xtxp8 | 2 | (TG)31 | ATATGGAAGGAAGAAGCCGG | AACACAACATGCACGCATG | 148 | 60 |
| Xtxp10 | 9 | (CT)14 | ATACTATCAAGAGGGGAGC | AGTACTAGCCACACGTCAC | 145 | 50 |
| Xtxp12 | 4 | (CT)22 | AGATCTGGCGGCAACG | AGTCACCCATCGATCATC | 193 | 55 |
| Xtxp13 | 2 | (TG)13 | TCTTTCCCAAGGAGCCTAG | GAAGTTATGCCAGACATGCTG | 120 | 55 |
| Xtxp14 | 5 | (GA)15 | GTAATAGTCATGACCGAGG | TAATAGACGAGTGAAAGCCC | 149 | 50 |
| Xtxp17 | 6 | (TC)16+(AG)12 | CGGACCAACGACGATTATC | ACTCGTCTCACTGCAATACTG | 164 | 55 |
| Xtxp19 | 2 | (AG)5+(AG)10 | CTTTCAATCGGTTCCAGAC | CTTCCACCTCCGTACTC | 206 | 55 |
| Xtxp20 | 10 | (AG)21 | TCTCAAGGTTTGATGGTTGG | ACCCATTATTGACCGTTGAG | 217 | 60 |
| Xtxp21 | 4 | (AG)18 | GAGCTGCCATAGATTTGGTCG | ACCTCGTCCCACCTTTGTTG | 179 | 60 |
| Xtxp32 | 1 | (AG)16 | AGAAATTCACCATGCTGCAG | ACCTCACAGGCCATGTCG | 133 | 60 |
| Xtxp34 | 3 | (CT)29 | TGGTTCGTATCCTTCTCTACAG | CATATACCTCCTCGTCGCTC | 365 | 55 |
| Xtxp43 | 1 | (CT)28 | AGTCACAGCACACTGCTTGTC | AATTTACCTGGCGCTCTGC | 171 | 60 |
| Xtxp48 | 3 | (GTCT)3+(ACG)3 | AATAACACGCGTCTAGTTGTC | CCATCATCGTCCATCC | 109 | 55 |
| Xtxp51 | 4 | (TG)11 | TCTCGGACTCAAGAGCAGAGG | GGACAGCAGCGGCTTCAG | 234 | 55 |
| Xtxp60 | 4 | (GT)4GC(GT)5 | GCTAGCTGACGCACGTCTCTG | TGCAACCGAGCGGTGACTA | 224 | 55 |

**Table 2** continued

| Marker | LG | Type of SSR(s) | Forward primer (5'to 3') | Reverse primer (5' to 3') | Size(bp) | T_a_ |
| --- | --- | --- | --- | --- | --- | --- |
| Xtxp65 | 5 | (ACC)4+(CCA)3CG(CT)8 | CACGTCGTCACCAACCAA | GTTAAACGAAAGGGAAATGGC | 128 | 55 |
| Xtxp67 | 9 | (GA)28 | CCTGACGCTCGTGGCTACC | TCCACACAAGATTCAGGCTCC | 175 | 55 |
| Xtxp75 | 1 | (TG)10 | CGATGCCTCGAAAAAAAAACG | CCGATCAGAGCGTGGCAGG | 172 | 50 |
| Xtxp88 | 1 | (AG)31 | CGTGAATCAGCGAGTGTTGG | TGCGTAATGTTCCTGCTC | 144 | 53 |
| Xtxp96 | 2 | (GA)24 | GCTGATGTCATGTTCCCTCAC | CATTCGTGGACTCTGTCGG | 198 | 52 |
| Xtxp123 | 5 | (AT)9 | TCGGCGAGCATCTTACA | TACGTAGGCGGTTGGATT | 279 | 55 |
| Xtxp141 | 10 | (GA)23 | TGTATGGCCTAGCTTATCT | CAACAAGCCAACCTAAA | 163 | 55 |
| Xtxp159 | 7 | (CT)21 | ACCCAAAGCCCAAATCAG | GGGGGAGAAACGGTGAG | 169 | 55 |
| Xtxp176 | 6 | (AG)4AAC(GA)4 | TGGCGGACATCCTATT | GGAGAGCCCGTCACTT | 161 | 55 |
| Xtxp177 | 4 | (CT)7(GT)8 | GCCGGTTGTGACTTG | TTAAAGCGATGGGTGTAG | 169 | 55 |
| Xtxp201 | 2 | (GA)36 | GCGTTTATGGAAGCAAAAT | CTCATAAGGCAGGACCAAC | 222 | 55 |
| Xtxp210 | 8 | (CT)10 | CGCTTTTCTGAAAATATTAAGGAC | GATGAGCGATGGAGGAGAG | 188 | 55 |
| Xtxp211 | 2 | (CT)23 | TCAACGGCCAATGATTTCTAAC | AGGTTGCGAATAAAAGGTAATGTG | 216 | 55 |
| Xtxp217 | 10 | (GA)23 | GGCCTCGACTACGGAGTT | TCGGCATATTGATTTGGTTT | 175 | 55 |
| Xtxp213 | 2 | (CT)27 | AACATTTCTTCCAGGCTC | GGAAGCAGTGCAGGAT | 168 | 50 |
| Xtxp230 | 9 | (GA)28 | GCTACCGCTGCTGCTCT | AGGGGGCATCCAAGAAAT | 191 | 55 |
| Xtxp248 | 1 | (AG)5(GA)28 | GGGTGTCCAATGTTGTCTGC | GGCCGTTACTGTCCCTTACTCA | 235 | 50 |

| Marker | LG | Type of SSR(s) | Forward primer (5'to 3') | Reverse primer (5' to 3') | Size (bp) | T_a_ |
| --- | --- | --- | --- | --- | --- | --- |
| Xtxp273 | 8 | (TTG)20 | GTACCCATTTAAATTGTTTGCAGTAG | CAGAGGAGGAGGAAGAGAAGG | 223 | 55 |
| Xtxp278 | 7 | (TTG)12 | GGGTTTCAACTCTAGCCTACCGAACTTCCT | ATGCCTCATCATGGTTCGTTTTGCTT | 249 | 50 |
| Xtxp285 | 3 | (CTT)11CTC(CTT)16 | ATTTGATTCTTCTTGCTTTGCCTTGT | TTGTCATTTCCCCCTTCTTTCTTTT | 249 | 55 |
| Xtxp295 | 7 | (TC)19 | AAATCATGCATCCATGTTCGTCTTC | CTCCCGCTACAAGAGTACATTCATAGCTTA | 165 | 55 |
| Xtxp297 | 2 | (AAG)24 | GACCCATATGTGGTTTAGTCGCAAAG | GCACAATCTTCGCCTAAATCAACAAT | 220 | 55 |
| Xtxp302 | 1 | (TGT)8 | TAGGTTCTGGACCACTTTTCTTTTTGTGTT | GAATCAACTATGTGCTTGCATTGTGCT | 180 | 55 |
| Xtxp303 | 5 | (GT)13 | AATGAGGAAAATATGAAACAAGTACCAA | AATAACAAGCGCAACTATATGAACAATAAA | 160 | 55 |
| Xtxp304 | 2 | (TCT)42 | ACATAAAAGCCCCTCTTC | CTTTCACACCCTTTATTCA | 206 | 55 |
| Xtxp317 | 6 | (CCT)5(CAT)11 | CCTCCTTTTCCTCCTCCTCCC | TCAGAATCCTAGCCACCGTTG | 162 | 55 |
| Xtxp319 | 1 | (TC)17 | TAGACATCTGAATTAAGGAGC | CATGCCCCTGAAAGAGA | 160 | 55 |
| Xtxp340 | 1 | (TAC)_15_ | AGAACTGTGCATGTATTCGTCA | AGAAACTCCAATTATCATCCATCA | 198 | 55 |
| Xtxp354 | 8 | (GA)21+(AAG)3 | TGGGCAGGGTATCTAACTGA | GCCTTTTTCTGAGCCTTGA | 157 | 55 |
| Xgap1 | 10 | (AG)16 | TCCTGTTTGACAAGCGCTTATA | AAACATCATACGAGCTCATCAATG | 260-300 | 60 |
| Xgap34 | 8 | (AC/CG)15 | AACAGCAGTAATGCCACAC | TGACTTGGTAGAGAACTTGTCTTC | 190 | 60 |
| Xgap42 | 1 | (AG)26 | TTTTCCTCTTTCAGATAACCGTA | CCCACCAAGGGCATC | 180 | 57 |

**Table 2** continued

LG = Linkage group; T_a_ = Annealing temperature

**Table 3** Mean squares for cold tolerance-related traits during early and normal planting regimes

| Early Planting | | | | | |
| --- | --- | --- | --- | --- | --- |
| Source | df | Seedling emergence (%) | Seedling vigor (1-5) | Seedling height (cm) | Seedling dry weight (g) |
| Environment (E) | 1 | 81238.44*** | 182.19*** | 33153.66*** | 1.79*** |
| Rep/E | 4 | 821.43 | 10.56 | 43.07 | 0.11 |
| Genotype (G) | 135 | 1240.85*** | 3.89*** | 38.12*** | 0.05*** |
| G x E | 135 | 357.15*** | 1.33** | 16.01*** | NS |
| Error | 540 | 82.34 | 0.71 | 5.33 | 0.02 |
| Normal planting | | | | | |
| Environment (E) | 1 | 8246.19*** | 4.94** | 12356.16*** | 8.43*** |
| Rep/E | 4 | 1060.76 | 3.03 | 41.65 | 4.21 |
| Genotype (G) | 135 | 1320.90*** | 1.92*** | 44.37*** | 1.77*** |
| G x E | 135 | 895.27*** | 1.13 | 11.98*** | 0.74* |
| Error | 540 | 157.79 | 0.55 | 3.67 | 0.78 |
| **,*** Significance at *p* < 0.01, 0.0001 | | | | | |

**Table 4** Pearson correlation (*r*) coefficients between early and normal planting regimes for seedling cold tolerance-related traits

|  |  | Early planting | | | |
| --- | --- | --- | --- | --- | --- |
| Normal planting | Trait | Seedling emergence (%) | Seedling vigor  (1-5) | Seedling height (cm) | Seedling dry weight  (g) |
|  | Seedling emergence (%) | 0.567*** | - | - | - |
|  | Seedling vigor (1-5) | - | 0.486*** | - | - |
|  | Seedling height (cm) | - | - | 0.389*** | - |
|  | Seedling dry weight(g) | -- | - | - | 0.542*** |

**Table 5** Pearson correlation coefficients of sorghum seedling traits under early and normal planting regimes at Manhattan, KS in 2010 and 2011

|  | | | Seedling emergence (%) | *^a^*Seedling vigor (1-5) | Seedling height (cm) | Seedling dry weight  (g) |
| --- | --- | --- | --- | --- | --- | --- |
| Early-planting | 2010 | Seedling emergence (%) | - |  |  |  |
|  |  | Seedling vigor (1-5) | -0.69*** | - |  |  |
|  |  | Seedling height (cm) | 0.60*** | -0.70*** | - |  |
|  |  | Seedling dry weight (g) | 0.61*** | -0.74*** | 0.70*** | - |
|  | 2011 | Seedling emergence (%) | - |  |  |  |
|  |  | Seedling vigor (1-5) | -0.73*** | - |  |  |
|  |  | Seedling height (cm) | 0.50*** | -0.73*** | - |  |
|  |  | Seedling dry weight (g) | 0.45*** | -0.60*** | 0.62*** | - |
| Normal planting | 2010 | Seedling emergence (%) | - |  |  |  |
|  |  | Seedling vigor (1-5) | -0.29** | - |  |  |
|  |  | Seedling height (cm) | NS | -0.43*** | - |  |
|  |  | Seedling dry weight (g) | 0.46*** | -0.65*** | 0.33** | - |
|  | 2011 | Seedling emergence (%) | - |  |  |  |
|  |  | Seedling vigor (1-5) | -0.34** | - |  |  |
|  |  | Seedling height (cm) | NS | -0.65*** | - |  |
|  |  | Seedling dry weight (g) | NS | -0.62*** | 0.80*** | - |

*^a^*Seedling vigor rated as 1 = excellent vigor and 5= poor vigor; NS means non-significance at *p*< 0.05; *, **,*** Significance at *p* < 0.05, 0.01, 0.0001, respectively

**Figure 1:** Frequency distribution of sorghum accessions for seedling emergence and vigor under early-planting

**Figure 2:** Frequency distribution of sorghum accessions for seedling emergence and height under normal planting

**Figure 3** Frequency distribution of accessions for seedling dry weight under normal planting
